# Development of a Clinical Risk Score for Prediction of Life-Threatening Arrhythmia Events in Patients with ST Elevated Acute Coronary Syndrome after Primary Percutaneous Coronary Intervention

**DOI:** 10.3390/ijerph19041997

**Published:** 2022-02-10

**Authors:** Thanutorn Wongthida, Lalita Lumkul, Jayanton Patumanond, Wattana Wongtheptian, Dilok Piyayotai, Phichayut Phinyo

**Affiliations:** 1 Office of Research and Knowledge Management, Chiang Rai Hospital, Chiang Rai 57000, Thailand; thanutorn.tw@gmail.com; 2 Center for Clinical Epidemiology and Clinical Statistics, Faculty of Medicine, Chiang Mai University, Chiang Mai 50200, Thailand; lalita.lumkul@gmail.com (L.L.); jpatumanond@gmail.com (J.P.); 3 Center of Multidisciplinary Technology for Advanced Medicine (CMUTEAM), Faculty of Medicine, Chiang Mai University, Chiang Mai 50200, Thailand; 4 Cardiology Unit, Department of Medicine, Chiang Rai Hospital, Chiang Rai 57000, Thailand; wwongtheptien@yahoo.com; 5 Cardiology Unit, Department of Medicine, Faculty of Medicine, Thammasat University, Pathum Thani 10120, Thailand; dilokp@tu.ac.th; 6 Department of Family Medicine, Chiang Mai University, Chiang Mai 50200, Thailand; 7 Musculoskeletal Science and Translational Research (MSTR), Chiang Mai University, Chiang Mai 50200, Thailand

**Keywords:** risk assessment, ST Elevation Myocardial Infarction, primary percutaneous coronary intervention, life threatening arrhythmia

## Abstract

ST-elevated acute coronary syndrome (STEACS) is a serious condition requiring timely treatment. Reperfusion with primary percutaneous coronary intervention (pPCI) is recommended and preferred over fibrinolysis. Despite its efficacy, lethal complications, such as life-threatening arrhythmia (LTA), are common in post-PCI patients. Although various risk assessment tools were developed, only a few focus on LTA prediction. This study aimed to develop a risk score to predict LTA events after pPCI. A risk score was developed using a retrospective cohort of consecutive patients with STEACS who underwent pPCI at Chiangrai Prachanukroh Hospital from January 2012 to December 2016. LTA is defined as the occurrence of malignant arrhythmia that requires advanced cardiovascular life support (ACLS) within 72 h after pPCI. Logistic regression was used for model derivation. Among 273 patients, 43 (15.8%) developed LTA events. Seven independent predictors were identified: female sex, hemoglobin < 12 gm/dL, pre- and intra-procedural events (i.e., respiratory failure and pulseless arrest), IABP insertion, intervention duration > 60 min, and desaturation after pPCI. The LTA score showed an AuROC of 0.93 (95%CI 0.90, 0.97). The score was categorized into three risk categories: low (<2.5), moderate (2.5–4), and high risk (>4) for LTA events. The LTA score demonstrated high predictive performance and potential clinical utility for predicting LTA events after pPCI.

## 1. Introduction

ST elevated acute coronary syndrome (STEACS) is a life-threatening condition requiring immediate and effective interventions to achieve favorable treatment outcomes. Currently, coronary artery reperfusion therapy with either primary percutaneous coronary intervention (pPCI) or fibrinolytic therapy is recommended in all patients with STEACS as it significantly outperformed non-reperfusion therapy in terms of mortality [1,2]. In addition, several comparative studies of pPCI and fibrinolysis therapy have proven that pPCI provided better clinical outcomes in terms of major adverse cardiac events, rate of heart failure, mechanical complications, and cardiac arrest [3,4]. Therefore, in most cases of STEACS, pPCI is a treatment of choice, especially if it can be performed in a timely manner within 120 min of first medical contact. Despite its superior efficacy and progressive improvements in cardiac care, a remarkable number of complications were still observed in patients with STEACS who underwent pPCI, and the in-hospital mortality was estimated from 1.6% to 13.8% [5,6].

Life-threatening arrhythmia (LTA) is one of the major post-procedural complications in patients with STEACS who underwent pPCI. The incidence of LTA was variedly reported from 2.0% to 15.7%, depending on the study populations [6,7]. It encompasses several lethal arrhythmic phenomena, such as sustained ventricular tachycardia (VT), ventricular fibrillation (VF), pulseless electrical activity (PEA), and asystole. All of which need emergent advanced cardiac life support (ACLS) and are associated with a substantial risk of death [6,7,8]. To date, several risk factors contributing to LTA in pPCI-treated patients were reported in several categories, such as the patient age, underlying diseases (e.g., chronic kidney disease), clinical presentation (e.g., Killip classification III or IV), vital signs (e.g., lower baseline heart rate), initial findings in electrocardiogram or echocardiography (e.g., baseline ST deviation, lower ejection fraction (EF)), initial laboratory investigations (e.g., lower hematocrit, higher white blood cell count, higher baseline serum creatinine), findings from coronary angiogram (CAG), and pPCI (e.g., pre PCI thrombolysis in myocardial infarction (TIMI) flow grade, ST resolution, post PCI TIMI flow grade) [7,8]. Interestingly, there was heterogeneity among past studies regarding the follow-up period, ranging from 12 h to even a week after pPCI [6,7,8].

To apply these predictors in practice, clinicians should consider these predictors simultaneously in a multivariable fashion to provide a more individualized prediction of the possibility for LTA, so optimal management could be undertaken for each patient [9]. This is often done by developing a multivariable prediction tool. Although several risk assessment tools have been developed for risk stratification in patients with STEACS (e.g., GRACE score [10], CRUSADE score [11]), most did not directly predict the occurrence of LTA. In 2019, one study was conducted in China to develop a risk assessment tool to predict VT or VF based on eight clinical predictors [12]. This simple scoring scheme classifies patients with STEACS who underwent pPCI into four risk grading with an outstanding discriminative ability at AuROC 0.9. However, the score was developed only to predict the occurrence of VT or VF and did not consider other types of arrhythmias that also require ACLS. Moreover, the prediction was limited within the 48-h time frame. Given that early VT in post-acute myocardial infarction mostly occurred within 48–72 h after pPCI [1,13], identifying lethal cardiac events during 72 h might be more appropriate. The present study aimed to develop a clinical prognostic score to predict LTA within 72 h after pPCI in patients with STEACS.

## 2. Materials and Methods

### 2.1. Study Design and Patients

A clinical risk score was developed with a retrospective observational cohort design. This study included a consecutive series of patients with STEACS who underwent pPCI at Chiangrai Prachanukroh Hospital from January 2012 to December 2016. We excluded patients who did not survive pPCI. The study protocol was approved by the Ethics Committee in Human Research of Chiangrai Prachanukroh Hospital (Certificate of Approval Ref.no.CR 0032.102/EC 271).

### 2.2. Setting and Procedure

Chiangrai Prachanukroh Hospital is a 758-bed tertiary care center with a specialized cardiology unit and catheterization laboratory. According to the unpublished statistical reports of our cardiac catheterization laboratory, the average number of pPCI cases and PCI cases from 2012 to 2016 were 55 and 357, respectively. During this period, there were four cardiac interventionists. Our institution used single-plane fluoroscopy for performing CAG and PCI. Types of contrast media used depended on the renal function of the patients (Iopromide (Ultravist) for patients with preserved renal function and Iodixanol (Visiplaque) for patients with impaired renal function). Only drug-eluting stents (DES) and bare-metal stents (BMS) were used in our center during the study period.

### 2.3. Data Collection

All the data used in the analysis were retrieved from the electronic medical record and standardized routine record forms for patients with STEACS. Baseline clinical characteristics were collected: age, sex, comorbidity, previous history of CAG and PCI, initial laboratory investigation. The data on pre- or intra-procedural cardiac events, including cardiogenic shock, respiratory failure, heart failure, and pulseless arrest, were collected. CAG and pPCI characteristics were also reviewed and extracted. After being transferred back to the CCU, initial vital signs were collected.

### 2.4. Candidate Predictors and Definitions of Predictors

As the evidence regarding the independent predictors of LTA in patients with STEACS was not solidified, all collected variables in our study were explored for their statistical significance and were considered as candidate predictors for the score development. Continuous predictors were categorized based on specified cutoff points from previous studies to prevent data-driven selection of cutoff points that would lead to overfitting. The details on the categorization of continuous predictors and their references are provided in Appendix A (Table A1). Only predictors with statistical significance from univariable analysis were included as candidate predictors in model development.

In this study, cardiogenic shock was defined as patients with systolic blood pressure <90 mmHg administered with vasopressors or inotropes. In our study, the definition of cardiogenic shock was equivalent to that of Killip classification IV [14]. Heart failure was described as shortness of breath, orthopnea, basal rales on lung auscultations, radiographic signs of pulmonary congestion, and administration of diuretic. The definition of heart failure in our study was equivalent to that of Killip classification II or III [14], depending on the severity. Respiratory failure was determined by shortness of breath or dyspnea that required endotracheal intubation with mechanical ventilation. A pulseless arrest was determined in pulseless patients who required ACLS. For these patients, the cardiac rhythm could be any of the following: VT, VF, PEA, or asystole. As patients with pre- or intraprocedural fatal arrhythmia or pulseless were usually prescribed antiarrhythmic agents, inotropic drugs, and vasopressors, we did not consider these drugs as candidate predictors to avoid clinical and statistical collinearity. 

### 2.5. Endpoint of Interest

The endpoint for prediction was the occurrence of LTA, including VT, VF, PEA, or asystole, which require ACLS within 72 h after pPCI. The presence of pulseless arrest before or during pPCI would be considered as a predictor not an outcome. 

### 2.6. Study Size Estimation

We based our study on the data from previous risk assessment by Huang et al. [12] and our previous report on unexpected events after pPCI [6] to estimate the minimum study size required for developing a multivariable prediction model for binary outcomes [15]. Providing that the expected AuROC was 0.90 with 10 candidate predictors, and the incidence of endpoint was approximately 14%, we calculated the Cox-Snell R-squared at 0.2709 [16]. The minimum number of events that would fulfill the three criteria: (1) a small overfitting, (2) a small absolute difference of 0.05 in the model’s apparent and adjusted R-squared value, and (3) a precise estimation, was 43 LTA events. Assuming the LTA incidence of 14%, 304 patients were required. 

### 2.7. Statistical Analysis

All statistical analyses were performed with Stata 17 (StataCorp, College Station, TX, USA). Based on their distribution, numerical data were described with mean and standard deviation (SD) or median and interquartile range (IQR). Categorical data were described with frequency and percentage. Univariable logistic regression was used to identify the unadjusted effect and the statistical significance of each potential predictor on the occurrence of LTA.

### 2.8. Multivariable Modeling and Score Development

Candidate predictors with significant *p*-values from univariable logistic regression were subsequently included in the multivariable logistic regression to derive the full model. Age and sex were forced into a multivariable model as these predictors were clinically important. Backward elimination of non-significant predictors was then conducted in a stepwise manner. The decision to eliminate each predictor was based on the magnitude of association (the size of the odds ratio), the statistical significance (the *p*-values), and the decrement of an area under the receiver operating characteristic curve (AuROC). After model reduction, the regression coefficients in their log-odds form of the remaining predictors were determined and used for generating the weighted score. The coefficient of each predictor was divided by the minimum coefficient among them. The division products were rounded up to the closest integer values and summed up to yield the total score for the prediction of LTA.

### 2.9. Test of Score Performance

The score performance was assessed in terms of discrimination, calibration, and clinical utility [17]. AuROC was used to evaluate the discriminative ability of the derived score. The calibration curve and Hosmer–Lemeshow goodness-of-fit test were used to assess calibration. The potential clinical utility of the score was determined using decision curve analysis (DCA) [18], which calculates the net benefit (NB) of using the score in practice to classify patients across a range of clinically relevant threshold probability in comparison to two default strategies of treating all or not treating any patients. Internal validation was performed with a bootstrap re-sampling procedure at 1000 replicates to evaluate the optimism of the model.

### 2.10. Score Classification

The scores were classified into three risk groups for clinical applicability: low risk, moderate risk, and high risk. The selection of cutoff points for score classification was chosen based on group-specific likelihood ratios (LR) of LTA. For the low-risk group, lower cutoff points were chosen to minimize the magnitude of LR, whereas, for the high-risk group, higher cutoff points were chosen to maximize the magnitude of LR. The LR of the moderate-risk group was determined to be close to 1.0. The discriminative ability of the score after classification was re-assessed by observing whether the 95%CIs of group-specific LR were overlapped with one another.

## 3. Results

**Figure 1:**
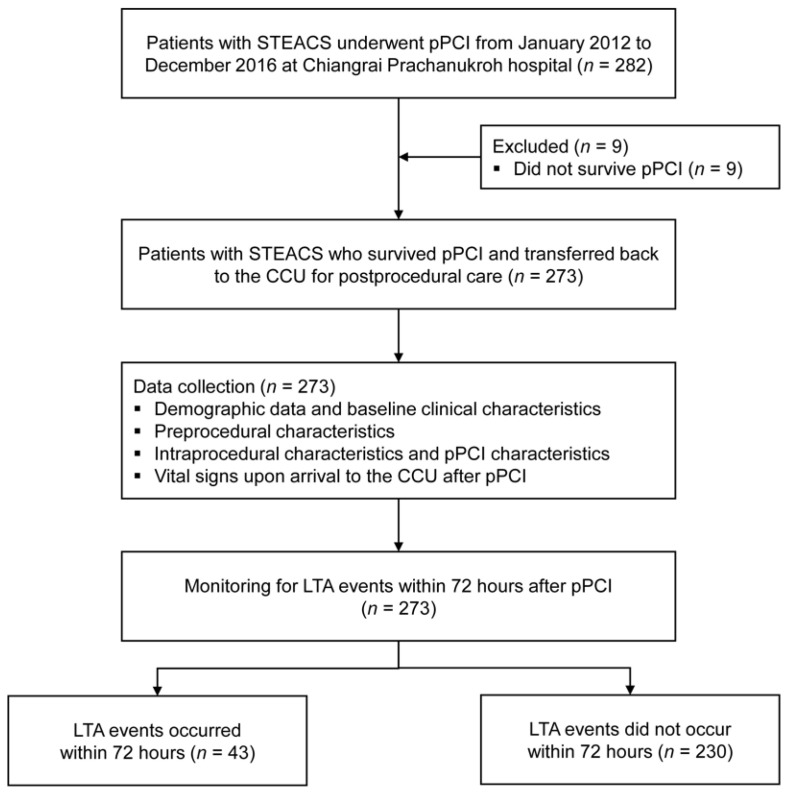
Study flow diagram of the patient cohort.

From January 2012 to December 2016, there were 282 patients STEACS who underwent pPCI at our institute. Of these, we excluded nine patients who did not survive pPCI. Among the remaining 273 patients, there were 43 patients (15.8%) with LTA events within 72 h after pPCI (Figure 1). Two-thirds of the included patients were male (169, 61.9%) with a mean age of 64.3 ± 12.7 years. Almost all patients (266 patients, 97.4%) had no previous history of either CAG or pPCI before this index pPCI. The median onset-to-balloon time was 318.5 min (IQR 237, 491). Table 1 summarizes the significant prognostic characteristics of LTA event within 72 h after pPCI. The details on the remaining prognostic characteristics, including the types of stents used, mode of access, the medication used during the periprocedural period, and the timing of pPCI, are shown in Appendix A (Table A2 and Table A3).

All significant predictors from the univariable analysis presented in Table 1 were included in multivariable analysis. The model reduction was then conducted in a stepwise manner, as described in the methods section. The final predictors for LTA event included female sex, hemoglobin < 12 gm/dL, pre- or intraprocedural respiratory failure, pre- or intraprocedural pulseless arrest, intervention time > 60 min, IABP insertion, and oxygen saturation at CCU < 94% (Table 2). The reduced multivariable model showed an outstanding AuROC of 0.94 (95%CI 0.90, 0.97).

**Figure 2:**
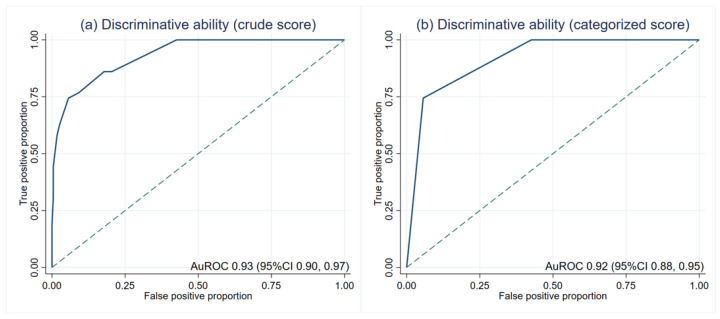
Discriminative ability based on the area under the receiver operating characteristic curve (AuROC). (**a**) crude score (**b**) categorized score.

The average scores in patients with and without LTA events were significantly different (5.3 ± 1.7 and 1.7 ± 1.5, *p* < 0.001). The crude score showed an outstanding discriminative ability at an AuROC of 0.93 (95%CI 0.90, 0.97) (Figure 2a). Then, the risk scores were categorized into low, moderate, and high-risk (Table 3). The LR was 13.17 (95%CI 7.55, 22.97) in the high-risk group. The LR of the low-risk group was lower than zero as no patients in this group had LTA events. There was no overlapping between the LR of each category, which indicates the discriminative ability of the categorized score. After score categorization, the AuROC showed a minimal drop to 0.92 (95%CI 0.88, 0.95) (Figure 2b).

**Figure 3:**
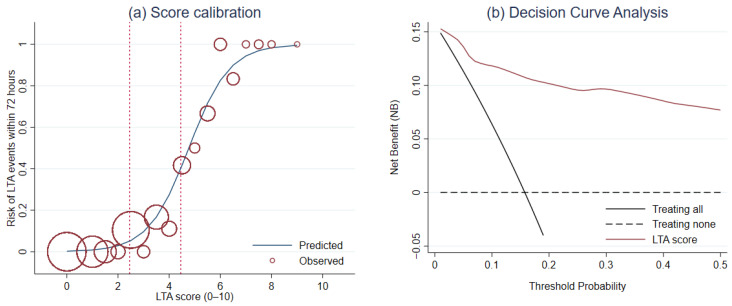
The evaluation of the score performance in terms of (**a**) calibration and (**b**) clinical usefulness using the score calibration curve and the decision curve analysis, respectively.

For calibration, Hosmer–Lemeshow goodness-of-fit test was not significant (*p* = 0.564). The score calibration plot was visualized (Figure 3a). DCA revealed a greater NB of LTA risk score than the NB of the default strategies across a range of threshold probability (Figure 3b). 

We conducted an interval validation with the bootstrapping procedure to evaluate the model optimism. The apparent AuROC was estimated at 0.942 (SD 0.016), whereas the test AuROC was estimated at 0.929 (SD 0.006). The estimated optimism of AuROC was +0.013 (SD 0.017).

## 4. Discussion

This study developed a new prognostic score for LTA in patients with STEACS who underwent pPCI. The LTA score predicts the probability of LTA within 72 h after pPCI based on seven readily available clinical predictors, including sex, initial hemoglobin level, pre- and intra- procedural respiratory failure, pre- and intra- procedural pulseless arrest, insertion of IABP, duration of intervention, and initial oxygen saturation at CCU. The score showed outstanding discriminative ability in predicting LTA events. Applying the score to patients with STEACS who are transferred back to the CCU after pPCI might benefit attending nurses for risk stratification and planning of care during the post-procedural period.

Indeed, many prediction rules have been developed to aid clinical decision makings in the care of patients with STEACS, such as GRACE score [10], CADILLAC risk score [19], PAMI score [20], TIMI [21], dynamic TIMI [22], and Zwolle risk score [23]. Most of them consider mortality and MACE as the endpoint of interest, and the timing of these endpoints could be from as short as 30 days or up to 3 years [21,23]. Therefore, it might be difficult for attending health care providers to apply these tools for predicting post-procedural complications or in-hospital outcomes. However, the evidence regarding these short-term predictions in patients with STEACS is still limited. One scoring scheme was recently developed to predict the occurrence of VT or VF [12]. However, the study only considered the event up to 48 h after pPCI. Based on previous studies, some LTA events can occur after this time point and may extend up to 72 h [6,13]. Therefore, in this study, we developed a score for LTA prediction within 72 h after pPCI. We extended the definition for LTA by including LTA other than VT or VF, such as PEA and asystole, as these events also require ACLS.

Seven independent predictors were used to develop the LTA score from the multivariable analysis. Most of the predictors within the score were previously reported to be associated with unfavorable outcomes in patients with STEACS, such as in-hospital mortality, which may partly be attributed to LTA. Being female has been identified as a significant predictor of mortality in several studies [24]. Higher incidence of VF and anterior myocardial infarction were reported in females, which undoubtedly increased the risk of in-hospital mortality [25]. In another study of out-of-hospital sudden cardiac arrest, females showed a significant association with PEA [26]. Anemia, or low baseline hemoglobin, in patients with ACS was also another indicator of poor clinical outcomes and higher mortality rates [27]. In addition, baseline anemia might be a surrogate for several conditions that affect the prognosis of patients with STEACS [27], such as malignancies and inflammatory disorders.

Oxygen desaturation at CCU was one of the strongest predictors in our score, which might indicate ongoing hypoxemia after pPCI. Some experimental studies have identified the potential association between desaturations and the occurrence of ventricular arrhythmias. However, the underlying mechanism was still unclear [28]. Patients with respiratory failure were found to be at higher risk of LTA. The requirement for endotracheal intubation was mainly due to acute pulmonary congestion. The previous study has shown that intubated patients with ACS who underwent pPCI had a higher in-hospital mortality rate than non-intubated patients [29]. The worsening of in-hospital prognosis in intubated patients can result from both the pathophysiologic changes due to intubation and ventilation [30] and the severity of acute heart failure and myocardial infarction. Pre- and intra-procedural pulseless arrest also has a major impact on myocardial ischemia and usually is accompanied by aggressive ventricular arrhythmia and recurrent cardiac arrest. It was reported that ACS patients with pulseless arrest or cardiogenic shock before pPCI had significantly higher in-hospital mortality [31]. These patients often require the insertion of IABP to stabilize their circulation. Both IABP insertion and prolonged intervention time indicate the severity of the patients in several aspects, such as the complexity of the culprit lesion or clinical instability, which required additional invasive interventions [31,32].

Several known factors were associated with mortality and treatment outcomes in STEMI patients but were not included in our score. The type of stents used during an intervention had been found to be associated with long-term outcomes and mortality in patients with STEMI [33]. Although the different types of cardiac intervention did not show the statistical significance and were not included in our scoring, there was a trend that patients with DES were less likely to have LTA events than patients with BMS. The mode of approach for pPCI was another important aspect to consider. Radial access was associated with lower periprocedural mortality and access site complications than femoral access and, thus, should be encouraged if experienced operators were available [34,35]. In this study, only 5% of the patients had pPCI performed through radial access, and none of these patients had LTA events. Due to the lower proportion of radial access performed, it was unlikely that the score performance would be affected. However, the generalizability of the score should indeed be limited to femoral access. Finally, the timing of PCI was another factor associated with periprocedural mortality. Our data showed a trend toward higher LTA events for pPCI cases performed off-hours (04.01 PM to 00.00 AM) than cases performed on-hours (06.00 AM to 04.00 PM), which was in agreement with the recent study [36]. The presence of these factors outside of our scoring components should not be overlooked and should be used inclusively during clinical decision making. 

According to the previous risk assessment tool, age, diabetes mellitus, serum potassium, heart rate, ST-segment elevation characters, left ventricular ejection fraction (LVEF), and TIMI flow grade were included to predict the occurrence of VT or VF [12]. Obviously, all of the predictors used in the previous study were not used in our study. Some of the predictors were included in our analysis but were removed from the model during backward elimination due to statistical insignificance. The initial selection of candidate predictors heavily influenced the difference during statistical modeling and the list of final predictors. In this study, we selected the routinely measured clinical parameters to maximize the applicability of the score, such as initial laboratory investigations, pre- and intra-procedural cardiopulmonary events, and initial vital signs at CCU. Our predictors also encompass the use of life-support equipment, which is an apparent bedside parameter that requires no further investigation or subjective interpretation. In comparison to the previous risk model, the newly developed LTA score showed comparable performance in discriminating patients with and without LTA (AuROC 0.90 vs. 0.93). 

The LTA score was intended to be used mainly by the intensive cardiac care nurses or cardiologists who need to attend and monitor patients with STEACS who recently underwent pPCI. For practical reasons, the score was classified into three risk groups based on the predicted probability of LTA: low (<2.5), moderate (2.5–4), and high risk (>4). In this study, no patients in the low-risk group experienced LTA during their hospital stay. Therefore, the likelihood of LTA was significantly below zero. For these low-risk patients, standard post-procedural monitoring is adequate. Transferring these low-risk patients to general medical wards or referring them back to their catchment hospitals for the continuation of care might be safe, especially when the ICUs are overcrowded with higher-risk patients. On the contrary, patients with LTA scores >4 carried a significantly high risk of LTA. These patients should be closely monitored or placed close to the nurse station. The team should also be cautiously prepared and equipped for performing ACLS. For moderate-risk patients, standard monitoring is suggested. However, consideration of transferring to general wards or referring back might not be entirely safe until after 72 h of event-free monitoring.

Overall, the newly developed LTA score illustrated an outstanding ability in predicting patients with STEACS who were at high risk of LTA by using only seven routinely available objective clinical predictors. The LTA score also had a longer duration of follow-up time and considered a broader spectrum of LTA than the previous tool. In addition, the score also classified patients into three risk groups with directive managements specified for each one. With a non-binary classification of risk groups, health care providers could better prioritize the patients according to their risk of LTA. However, some limitations needed to be addressed. First, the number of candidate predictors with statistical significance was higher than expected; thus, the number of events available might not be sufficient to prevent model overfitting. However, based on bootstrapped internal validation, the degree of optimism was low, and the model still carried an outstanding performance. Nonetheless, validation of the score in samples with higher LTA events is suggested. Second, owing to the retrospective nature of data collection, the presence of some information bias might be unavoidable. However, as the endpoint of interest was objective, the magnitude of bias might be modest. Third, the data of the predictors used in the previous model by Huang et al. was routinely collected and not available in our setting [12], such as ST-segment elevation characters and LVEF. Thus, we could not include these predictors within our model or perform a comparative validation in terms of performance. Fourth, the data used was collected from 2012 to 2016, which is relatively old. Nonetheless, as there were no significant changes in our practice and local guidelines (i.e., treatment protocol or type of stent) from 2016 to 2021, we believe that our data is still generalizable to the current time. However, as pPCI was performed through femoral access in about 95% of the patients in this study, our scoring might be generalizable only to pPCI with femoral access. Finally, this study was based only on one tertiary care center, which limited the generalizability of the LTA score to other healthcare settings. Hence, it is recommended to conduct a prospective external validation study with a larger sample size before implementing this score in clinical practice. In addition, further study should examine the robustness of our scoring system in settings where radial access was mainly used.

## 5. Conclusions

Predicting the probability of LTA in patients with STEACS after pPCI might be beneficial to attending critical care nurses and cardiologists to plan optimal post-procedural monitoring and reduce in-hospital mortality. In this study, we developed the LTA score, which includes seven readily available clinical parameters: female, baseline hemoglobin, pre- and intra-procedural respiratory failure, pre- and intra-procedural pulseless arrest, intervention time, insertion of IABP, and initial desaturation at CCU. The score classified patients into three risk categories: low, moderate, and high risk, where patients with high risk should be provided with intensive monitoring, and the team should be prepared for ACLS. In this development dataset, the LTA score showed outstanding discriminative ability and good calibration for predicting LTA during the post-procedural period. External validation of the score is warranted to confirm the robustness of the score performance in other contexts.

## Figures and Tables

**Table 1:** Prognostic characteristics for life-threatening arrhythmia (LTA) within 72 h after primary PCI under univariable analysis.

Prognostic Characteristic	Missing Data	LTA within 72 h (*n* = 43, 15.8%)		No LTA within 72 h (*n* = 230, 84.2%)		Univariable Analysis		
	*n* (%)	*n*	(%)	*n*	(%)	uOR	95%CI	*p*-Value
Age (year, Mean ± SD)	0 (0)	67.1	(±10.9)	63.8	(±13.0)	1.02	0.99, 1.05	0.117
<60		10	(23.3)	70	(30.4)	1.00	Reference	
60–79		27	(62.8)	135	(58.7)	1.40	0.64, 3.06	0.398
≥80		6	(13.9)	25	(10.9)	1.68	0.55, 5.10	0.360
Female	0 (0)	21	(48.8)	83	(36.1)	1.69	0.88, 3.26	0.117
Atrial fibrillation	0 (0)	3	(7.0)	3	(1.3)	5.68	1.11, 29.12	0.037
Laboratory Investigation								
Hemoglobin < 12 gm/dL	0 (0)	30	(69.8)	86	(37.4)	3.86	1.91, 7.81	<0.001
Platelet count (cell/mm^3^)								
<150,000	0 (0)	11	(25.6)	16	(7.0)	4.66	1.98, 10.95	<0.001
150,000–449,000		31	(72.1)	210	(91.3)	1.00	Reference	
>449,000		1	(2.3)	4	(1.7)	1.69	0.18, 15.65	0.642
INR >1.8	3 (1.1)	4	(9.5)	2	(0.9)	11.90	2.11, 67.22	0.005
Creatinine > 1.5 mg/dL	0 (0)	18	(41.9)	44	(19.2)	3.04	1.53, 6.06	0.002
Bicarbonate (mmol/L)								
<22	0 (0)	36	(83.7)	115	(50.0)	5.10	2.18, 11.93	<0.001
22–30		7	(16.3)	114	(49.6)	1.00	Reference	
>30		0	(0)	1	(0.4)	NE	NE	
Calcium (mg/dL)								
<8.7	5 (1.8)	21	(51.2)	77	(33.9)	2.12	1.08, 4.19	0.030
8.7–10.2		19	(46.3)	148	(65.2)	1.00	Reference	
>10.2		1	(2.5)	2	(0.9)	3.90	0.34, 45.02	0.276
Magnesium (mg/dL)								
<1.5	5 (1.8)	0	(0)	7	(3.1)	NE	NE	
1.5–2.3		31	(75.6)	195	(85.9)	1.00	Reference	
>2.3		10	(24.4)	25	(11.0)	2.52	1.10, 5.74	0.028
CBG (mg%)								
<80	3 (1.1)	4	(9.8)	5	(2.2)	7.16	1.76, 29.08	0.006
80–180		18	(43.9)	161	(70.3)	1.00	Reference	
>180		19	(46.3)	63	(27.5)	2.70	1.33, 5.47	0.006
Pre- or intraprocedural events								
Cardiogenic shock	0 (0)	41	(95.4)	114	(49.6)	20.86	4.93, 88.28	<0.001
Respiratory failure	0 (0)	31	(72.1)	43	(18.8)	11.24	5.34, 23.65	<0.001
Heart failure	0 (0)	11	(25.6)	27	(11.7)	2.59	1.17, 5.72	0.019
Pulseless arrest	0 (0)	25	(58.1)	32	(14.0)	8.59	4.22, 17.51	<0.001
CAG and pPCI characteristics								
Intervention time > 60 min	0 (0)	28	(65.1)	56	(24.4)	5.80	2.89, 11.63	<0.001
Culprit lesion > 1 vessels	0 (0)	31	(72.1)	119	(51.7)	2.41	1.18, 4.92	0.016
IABP insertion	0 (0)	15	(34.9)	6	(2.6)	20.00	7.18, 55.74	<0.001
Contrast media used >100 ml	0 (0)	18	(41.9)	54	(23.5)	2.35	1.19, 4.62	0.014
Vital signs at CCU								
HR (/min)								
<60	0 (0)	4	(9.3)	17	(7.4)	1.82	0.56, 5.91	0.322
60–100		21	(48.8)	162	(70.4)	1.00	Reference	
>100		18	(41.9)	51	(22.2)	2.72	1.35, 5.50	0.005
SBP (mmHg)								
<90	0 (0)	10	(23.3)	9	(3.9)	7.48	2.78, 20.13	<0.001
90–140		26	(60.5)	175	(76.1)	1.00	Reference	
>140		7	(16.2)	46	(20.0)	1.02	0.42, 2.51	0.958
DBP (mmHg)								
<60	0 (0)	17	(39.5)	46	(20.0)	2.73	1.33, 5.60	<0.001
60–90		21	(48.8)	155	(67.4)	1.00	Reference	
>90		5	(11.7)	29	(12.6)	1.27	0.44, 3.65	0.654
Oxygen saturation <94%	0 (0)	18	(41.9)	14	(6.1)	11.11	4.93, 25.02	<0.001

Abbreviations: CAG, coronary angiogram; CBG, capillary blood glucose; CCU, cardiac care unit; CI, confidence interval; CK-MB, creatinine kinase-isoenzyme MB; DBP, diastolic blood pressure; HR, heart rate; IABP, intra-aortic balloon pump; INR, international normalize ratio; LTA, life-threatening arrhythmia; NE, not estimable; pPCI; primary percutaneous coronary intervention; SBP, systolic blood pressure; SD, standard deviation; TIMI, thrombolysis in myocardial infarction; uOR, univariable odd ratio.

**Table 2:** Prognostic characteristics for life-threatening arrhythmia (LTA) within 72 h after primary percutaneous coronary intervention (pPCI) under multivariable analysis.

Predictors	mOR	95%CI	*p*-Value	Log Odds Coefficient	Score
Sex					
Male	1.00	reference	-	-	0
Female	3.64	1.17, 11.32	0.026	1.29	1
Hemoglobin (gm/dL)					
≥12	1.00	reference	-	-	0
<12	8.54	2.64, 27.59	<0.001	2.14	1.5
Pre- or intraprocedural respiratory failure					
No	1.00	reference	-	-	0
Yes	4.18	1.33, 13.11	0.014	1.43	1
Pre- or intraprocedural pulseless arrest					
No	1.00	reference	-	-	0
Yes	7.86	2.04, 30.26	0.003	2.06	1.5
Intervention time (minute)					
≤60	1.00	reference	-	-	0
>60	3.59	1.32, 9.74	0.012	1.28	1
IABP insertion					
No	1.00	reference	-	-	0
Yes	9.45	2.47, 36.11	0.001	2.25	2
Oxygen saturation at CCU (%)					
≥94	1.00	reference	-	-	0
<94	11.10	3.42, 35.97	<0.001	2.41	2

Abbreviations: CCU, cardiac care unit; CI, confidence interval; IABP, intra-aortic balloon pump; mOR, multivariable odds ratio.

**Table 3:** Score categorization and the likelihood ratio of life-threatening arrhythmia (LTA) within 72 h after primary percutaneous coronary intervention (pPCI).

Probability Categories	Score	LTA within 72 h (*n* = 43)		No LTA within 72 h (*n* = 230)		LR	95%CI	*p*-Value
		*n*	%	*n*	%			
Low	<2.5	0	0	132	57.4	0	0, 0.16	<0.001
Moderate	2.5–4	11	25.6	85	37.0	0.69	0.31, 1.45	0.306
High	>4	32	74.4	13	5.6	13.17	6.07, 29.36	<0.001
Mean (±SD)		5.3	(±1.7)	1.7	(±1.5)			<0.001

Abbreviations: CI, confidence interval; LTA, life-threatening arrhythmia; LR, likelihood ratio; SD, standard deviation.

## Data Availability

Not applicable.

## Appendix A

**Table A1:** References for cutoff points of continuous predictors.

Predictor	Cutoff Point	References
Hemoglobin	<12 gm/dL	Goldenberg I, Barsheshet A, Laish-Farkash A, Swissa M, Schliamser JE, Michowitz Y, et al. Anemia and the risk of life-threatening ventricular tachyarrhythmias from the Israeli implantable cardioverter defibrillator registry. Am J Cardiol. 2017;120(12):2187–92. [37]
Platelet count	<150,000 cell/mm^3^	Shiraishi J, Koshi N, Matsubara Y, Nishimura T, Ito D, Kimura M, et al. Effects of baseline thrombocytopenia on in-hospital outcomes in patients undergoing elective percutaneous coronary intervention. Intern Med. 2019;58(12):1681–8. [38]
INR	>1.8	Bashore TM, Balter S, Barac A, Byrne JG, Cavendish JJ, Chambers CE, et al. 2012 American college of cardiology foundation/society for cardiovascular angiography and interventions expert consensus document on cardiac catheterization laboratory standards update: a report of the American college of cardiology foundation task force on expert consensus documents developed in collaboration with the society of thoracic surgeons and society for vascular medicine. J Am Coll Cardiol. 2012;12;59(24):2221–305. [39]
Creatinine (Cr)	>1.5 mg/dL	Moscucci M, Kline-Rogers E, Share D, O’Donnell M, Maxwell-Eward A, Meengs WL et al. Simple bedside additive tool for predict of in-hospital mortality after percutaneous coronary interventions. Circulation. 2001;104(3):263–8. [5]
Chloride	102–109 mmol/L	Kratz A, Pesce MA, Basner RC, Andrew J. Einstein AJ. Laboratory values of clinical importance. In: Loscalzo J, editor. Harrison’s cardiovascular medicine. 2nd ed. New York: McGraw-Hill Education; 2013. p. 549–74. [40]
Carbon dioxide	22–30 mmol/L	Kratz A, Pesce MA, Basner RC, Andrew J. Einstein AJ. Laboratory values of clinical importance. In: Loscalzo J, editor. Harrison’s cardiovascular medicine. 2nd ed. New York: McGraw-Hill Education; 2013. p. 549–74. [40]
Calcium	8.7–10.2 mg/dL	Kratz A, Pesce MA, Basner RC, Andrew J. Einstein AJ. Laboratory values of clinical importance. In: Loscalzo J, editor. Harrison’s cardiovascular medicine. 2nd ed. New York: McGraw-Hill Education; 2013. p. 549–74. [40]
Magnesium	1.5–2.3 mg/dL	Kratz A, Pesce MA, Basner RC, Andrew J. Einstein AJ. Laboratory values of clinical importance. In: Loscalzo J, editor. Harrison’s cardiovascular medicine. 2nd ed. New York: McGraw-Hill Education; 2013. p. 549–74. [40]
CKMB	>25 U/L	Cabaniss CD. Creatine kinase. In: Walker HK, Hall WD, Hurst JW, editors. Clinical methods: the history, physical, and laboratory examinations. 3rd ed. Boston: Butterworths; 1990. chapter 32. [41]
CBG	80–180 mg%	Baker L, Maley JH, Arévalo A, DeMichele F 3rd, Mateo-Collado R, Finkelstein S, et al. Real-world characterization of blood glucose control and insulin use in the intensive care unit. Sci Rep. 2020;10(1):10718. [42]
Onset to balloon time	>240 min	Alsamara M, Degheim G, Gholkar G, Hiner E, Zughaib M. Is symptom to balloon time a better predictor of outcomes in acute ST-segment elevation myocardial infarction than door to balloon time? Am J Cardiovasc Dis. 2018;8(4):43–7. [43]
Intervention time	>60 min	Estimated from average intervention time at 55.8 ± 22.1 min
Contrast media used	>100 mL	Estimated from average volume of contrast media used at 92.1 ± 42.2
Oxygen saturation	<94%	O’Driscoll BR, Howard LS, Earis J, Mak V. BTS guideline for oxygen use in adults in healthcare and emergency settings. Thorax. 2017;72(Suppl 1):ii1-ii90. [44]

## Appendix B

**Table A2:** Prognostic characteristics for life-threatening arrhythmia (LTA) within 72 h after primary PCI in terms of underlying diseases, initial laboratory investigations, and pre- or intraprocedural events.

Prognostic Characteristic	Missing Data	LTA within 72 h (*n* = 43, 15.8%)		No LTA within 72 h (*n* = 230, 84.2%)		Univariable Analysis		
	*n* (%)	*n*	(%)	*n*	(%)	uOR	95%CI	*p*-Value
Underlying disease								
Atrial fibrillation	0 (0)	3	(7.0)	3	(1.3)	5.68	1.11, 29.12	0.037
Coronary artery disease	0 (0)	3	(7.0)	11	(4.8)	1.49	0.40, 5.59	0.552
Chronic kidney disease	0 (0)	8	(18.6)	23	(10.0)	2.06	0.85, 4.96	0.108
COPD	0 (0)	3	(7.0)	19	(8.3)	0.83	0.24, 2.95	0.777
Dilated cardiomyopathy	0 (0)	1	(2.3)	7	(3.0)	0.76	0.09, 6.33	0.798
Dyslipidemia	0 (0)	13	(30.2)	101	(43.9)	0.55	0.28, 1.12	0.098
Diabetic mellitus	0 (0)	14	(32.6)	50	(21.7)	1.74	0.85, 3.54	0.127
Gout	0 (0)	3	(7.0)	21	(9.1)	0.75	0.21, 2.62	0.648
Hypertension	0 (0)	24	(55.8)	118	(51.3)	1.20	0.62, 2.31	0.587
History of CVA	0 (0)	1	(2.3)	11	(4.8)	0.47	0.06, 3.77	0.480
History CAG	0 (0)	0	(0)	7	(3.0)	NE	NE	
History PCI	0 (0)	0	(0)	6	(2.6)	NE	NE	
Laboratory Investigation								
Hemoglobin < 12 gm/dl	0 (0)	30	(69.8)	86	(37.4)	3.86	1.91, 7.81	<0.001
Platelet count (cell/mm^3^)								
<150,000	0 (0)	11	(25.6)	16	(7.0)	4.66	1.98, 10.95	<0.001
150,000–449,000		31	(72.1)	210	(91.3)	1.00	Reference	
>449,000		1	(2.3)	4	(1.7)	1.69	0.18, 15.65	0.642
INR >1.8	3 (1.1)	4	(9.5)	2	(0.9)	11.90	2.11, 67.22	0.005
White blood cell count (/mm^3^)								
≤10,000	0 (0)	12	(27.9)	65	(28.3)	1.00	Reference	
>10,000		31	(72.1)	165	(71.7)	1.02	0.49, 2.10	0.962
Creatinine > 1.5 mg/dL	0 (0)	18	(41.9)	44	(19.2)	3.04	1.53, 6.06	0.002
Sodium (mmol/L)								
<136	0 (0)	14	(32.6)	108	(47.0)	0.54	0.27, 1.08	0.080
136–146		29	(67.4)	121	(52.6)	1.00	Reference	
>146		0	(0)	1	(0.4)	NE	NE	
Potassium (mmol/L)								
<3.5	0 (0)	18	(41.9)	102	(44.4)	1.00	0.51, 1.96	0.988
3.5–5.0		22	(51.2)	124	(53.9)	1.00	Reference	
>5.0		3	(6.9)	4	(1.70	4.23	0.89, 20.20	0.071
Chloride (mmol/L)								
<102	0 (0)	12	(27.9)	60	(26.1)	1.28	0.60, 2.71	0.528
102–109		24	(55.8)	153	(66.5)	1.00	Reference	
>109		7	(16.3)	17	(7.4)	2.63	0.99, 6.99	0.054
Carbon dioxide (mmol/L)								
<22	0 (0)	36	(83.7)	115	(50.0)	5.10	2.18, 11.93	<0.001
22–30		7	(16.3)	114	(49.6)	1.00	Reference	
>30		0	(0)	1	(0.4)	NE	NE	
Calcium (mg/dL)								
<8.7	5 (1.8)	21	(51.2)	77	(33.9)	2.12	1.08, 4.19	0.030
8.7–10.2		19	(46.3)	148	(65.2)	1.00	Reference	
>10.2		1	(2.5)	2	(0.9)	3.90	0.34, 45.02	0.276
Magnesium (mg/dL)								
<1.5	5 (1.8)	0	(0)	7	(3.1)	NE	NE	
1.5–2.3		31	(75.6)	195	(85.9)	1.00	Reference	
>2.3		10	(24.4)	25	(11.0)	2.52	1.10, 5.74	0.028
CK-MB > 25 U/L	11 (4.0)	32	(74.4)	154	(67.0)	1.77	0.77, 4.03	0.177
CBG (mg%)								
<80	3 (1.1)	4	(9.8)	5	(2.2)	7.16	1.76, 29.08	0.006
80–180		18	(43.9)	161	(70.3)	1.00	Reference	
>180		19	(46.3)	63	(27.5)	2.70	1.33, 5.47	0.006
Pre- or intraprocedural events								
Cardiogenic shock	0 (0)	41	(95.4)	114	(49.6)	20.86	4.93, 88.28	<0.001
Respiratory failure	0 (0)	31	(72.1)	43	(18.8)	11.24	5.34, 23.65	<0.001
Heart failure	0 (0)	11	(25.6)	27	(11.7)	2.59	1.17, 5.72	0.019
Pulseless arrest	0 (0)	25	(58.1)	32	(14.0)	8.59	4.22, 17.51	<0.001

Abbreviations: CAG, coronary angiogram; CBG, capillary blood glucose; COPD, chronic obstructive pulmonary disease; CI, confidence interval; CK-MB, creatinine kinase-isoenzyme MB; CVA, cerebrovascular accident; INR, international normalize ratio; NE, not estimable; pPCI; primary percutaneous coronary intervention; uOR, univariable odd ratio.

**Table A3:** Prognostic characteristics for life-threatening arrhythmia (LTA) within 72 h after primary percutaneous coronary intervention (pPCI) in terms of coronary angiogram (CAG) and pPCI characteristics.

Prognostic Characteristic	Missing Data	LTA within 72 h (*n* = 43, 15.8%)		No LTA within 72 h (*n* = 230, 84.2%)		Univariable Analysis		
	*n* (%)	*n*	(%)	*n*	(%)	uOR	95%CI	*p*-Value
CAG & pPCI characteristics								
TIMI flow before pPCI								
0	0 (0)	40	(93.0)	190	(82.6)	1.00	Reference	
1		2	(4.7)	15	(6.5)	0.63	0.14, 2.88	0.554
2		1	(2.3)	24	(10.4)	0.20	003, 1.51	0.118
3		0	(0)	1	(0.5)	NE	NE	
TIMI flow after pPCI								
0	0 (0)	0	(0)	1	(0.4)	1.00	Reference	
1		1	(2.3)	0	(0)	NE	NE	
2		10	(23.3)	28	(12.2)	2.24	1.00, 5.06	0.051
3		32	(74.4)	201	(87.4)	NE	NE	
Onset to balloon time >240 min	1 (0.4)	33	(76.7)	162	(70.7)	1.37	0.64, 2.93	0.424
Mode of access								
Femoral access	0 (0)	43	(100.0)	217	(94.4)	1.00	Reference	
Radial access		0	(0)	13	(5.6)	NE	NE	
Intervention time >60 min	0 (0)	28	(65.1)	56	(24.4)	5.80	2.89, 11.63	<0.001
Culprit lesion >1 vessels	0 (0)	31	(72.1)	119	(51.7)	2.41	1.18, 4.92	0.016
Culprit lesion								
1 vessel	0 (0)	12	(27.9)	111	(48.2)	1.00	Reference	
2 vessels		19	(44.2)	71	(30.9)	2.48	1.13, 5.41	0.023
3 vessels		12	(27.9)	48	(20.9)	2.31	0.97, 5.51	0.059
pPCI Methods								
Aspiration	0 (0)	1	(2.3)	6	(2.6)	1.00	Reference	
Balloon		4	(9.3)	14	(6.1)	1.71	0.16, 18.73	0.659
Balloon & Aspiration		12	(27.9)	44	(19.3)	1.64	0.18, 14.93	0.662
Bare metal stents		6	(13.9)	8	(3.5)	4.50	0.42, 47.99	0.213
Bare metal stents & Aspiration		3	(7.0)	14	(6.1)	1.29	0.11, 15.00	0.841
Drug-eluting stents		11	(25.6)	70	(30.4)	0.94	0.10, 8.60	0.958
Drug-eluting stents & Aspiration		6	(14.0)	74	(32.2)	0.49	0.05, 4.73	0.535
IABP insertion	0 (0)	15	(34.9)	6	(2.6)	20.00	7.18, 55.74	<0.001
Contrast media used >100 ml	0 (0)	18	(41.9)	54	(23.5)	2.35	1.19, 4.62	0.014
Anti-thrombotic used *								
Heparin	0 (0)	15	(34.9)	72	(31.3)	1.00	Reference	
Eptifibatide		4	(9.3)	8	(3.5)	2.40	0.64, 9.01	0.195
Heparin and Eptifibatide		24	(55.8)	150	(65.2)	0.77	0.38, 1.55	0.462
Antiarrhythmic agents								
Amiodarone *	0 (0)	12	(27.9)	15	(6.5)	5.55	2.38, 12.95	<0.001
Adenosine *	0 (0)	4	(9.3)	20	(8.7)	1.08	0.35, 3.32	0.897
Atropine *	0 (0)	13	(30.2)	55	(23.9)	1.38	0.67, 2.83	0.381
Adrenaline *	0 (0)	23	(53.5)	24	(10.4)	9.87	4.74, 20.55	<0.001
Dopamine *	0 (0)	30	(69.8)	86	(37.4)	3.86	1.91, 7.81	<0.001
Norepinephrine*	0 (0)	16	(37.2)	20	(8.7)	6.22	2.88, 13.44	<0.001
Cardiologist								
A	0 (0)	12	(27.9)	51	(22.2)	1.00	Reference	
B		19	(44.2)	112	(48.7)	0.72	0.33, 1.60	0.420
C		1	(2.3)	10	(4.3)	0.43	0.05, 3.65	0.435
D		11	(25.6)	57	(24.8)	0.82	0.33, 2.02	0.666
Timing of pPCI								
06.00 AM to 04.00 PM	0 (0)	26	(60.5)	165	(71.7)	1.00	Reference	
04.01 PM to 00.00 AM		17	(39.5)	65	(28.3)	1.66	0.85, 3.26	0.141

Abbreviations: CI, confidence interval; IABP, intra-aortic balloon pump; NE, not estimable; pPCI; primary percutaneous coronary intervention; TIMI, thrombolysis in myocardial infarction; uOR, univariable odd ratio. * These factors were not considered as candidate predictors to avoid clinical and statistical collinearity with other prespecified predictors.
